# Antihypertensive treatment with hydrochlorothiazide-hydralazine combination aggravates medial vascular calcification in CKD rats with mineral bone disorder

**DOI:** 10.3389/fcvm.2023.1241943

**Published:** 2023-09-29

**Authors:** Richard Larivière, Roth-Visal Ung, Sylvain Picard, Darren E. Richard, Fabrice Mac-Way, Mohsen Agharazii

**Affiliations:** ^1^ Research Centre CHU de Québec, Endocrinology and Nephrology Axis, L'Hôtel-Dieu de Québec Hospital, Université Laval, Quebec, QC, Canada; ^2^ Department of Medicine, Faculty of Medicine, Université Laval, Quebec, QC, Canada; ^3^ Department of Molecular Biology, Medical Biochemistry, and Pathology, Faculty of Medicine, Université Laval, Quebec, QC, Canada

**Keywords:** chronic kidney disease, mineral bone disorder, vascular calcification, blood pressure, arterial stiffness, AT_1_ receptor antagonist, hydrochlorothiazide, hydralazine

## Abstract

**Background:**

Arterial stiffness and medial vascular calcification, leading to isolated systolic blood pressure (BP), are major cardiovascular risk factors in patients with chronic kidney disease (CKD) and mineral bone disorders (MBD). The impact of BP on MBD-induced medial vascular calcification in CKD remains uncertain. We investigated whether BP reduction improves arterial stiffness and medial vascular calcification in a rat model of CKD-MBD.

**Methods:**

CKD was induced in Wistar rats by subtotal nephrectomy. Then, MBD was generated by a Ca/P-rich diet with calcitriol supplementation to induce medial vascular calcification. Two antihypertensive treatments were evaluated: (1) the angiotensin AT_1_ receptor antagonist losartan, and (2) the combination of the thiazide diuretic hydrochlorothiazide and the direct vasodilator hydralazine (HCTZ/HY). After 5 weeks, mean BP (MBP), pulse pressure (PP), and pulse wave velocity (PWV) were determined. Vascular calcification was assessed in the thoracic aorta.

**Results:**

While MBP was similar in CKD-MBD and control CKD rats, PP and PWV were increased in CKD-MBD rats. The heightened arterial stiffness in CKD-MBD rats was associated with diffused medial calcification along the thoracic aorta. Although both losartan and HCTZ/HY reduced MBP in CKD-MBD rats, losartan did not affect PP and PWV nor medial vascular calcification, whereas HCTZ/HY, unexpectedly, further increased arterial stiffness and medial vascular calcification.

**Conclusion:**

In the rat model of CKD-MBD, antihypertensive treatment with losartan did not affect arterial stiffness or medial vascular calcification. However, HCTZ/HY treatment aggravated arterial stiffness and vascular calcification despite a similar reduction of MBP, suggesting a blood pressure-independent mechanism for vascular calcification.

## Introduction

Hypertension is a major risk factor for cardiovascular morbidity and mortality in patients with chronic kidney diseases (CKD) (1). Isolated systolic blood pressure, frequently observed in CKD patients, has been related to medial vascular remodeling and calcification, two conditions that significantly reduce arterial compliance (2). These maladaptive vascular changes lead to increased pulse pressure and pulse wave velocity, an index of arterial stiffness, which has been linked to excess cardiovascular mortality in CKD patients (3–5). Although there is a clear relationship between hypertension and arterial stiffness, the role of BP in medial vascular calcification remains uncertain.

While intimal calcification has been associated with plaque rupture in atherosclerosis, medial vascular calcification in CKD is related to Mönckeberg's sclerosis leading to arterial stiffness due, at least in part, to abnormal mineral metabolism including phosphate retention, a hallmark of mineral bone disorder (MBD) (6, 7). Increased phosphate levels were shown to be essential for vascular calcification and cardiovascular mortality in CKD patients (8–10). Hyperphosphatemia induces vascular smooth muscle cell differentiation into osteoblast-like cells leading to vessel mineralization. Vessel mineralization is associated with vascular smooth muscle marker loss, including α-smooth muscle actin, and *de novo* expression of bone-related proteins, including osteocalcin (11, 12). The loss of functional vascular smooth muscle cells was also associated with vascular remodeling, which includes elastic lamina disruption, and increased collagen synthesis and deposition (13). In addition, CKD-related medial vascular calcification has been linked to increased production of inflammatory cytokines, such as IL-6, IL-1β, and tumor necrosis factor, due, at least in part, to activated macrophage infiltration into the arterial wall (14).

We recently reported that endothelin-1 (ET-1) plays a key role in CKD-related medial vascular calcification. ET_A_ receptor blockade reduced BP and arterial stiffness together with medial vascular calcification in CKD rats with MBD (15). Although the protective effect of ET_A_ receptor blockade on vascular calcification was likely due to a direct inhibitory effect on mechanisms related to vascular remodeling, inflammation, and smooth muscle cell differentiation into osteoblast-like cells, we could not exclude that changes in BP were involved in the development of calcification.

To determine the role of BP on arterial stiffness and medial vascular calcification in CKD-MBD conditions, we investigated the effects of two antihypertensive treatments that have been widely used in animal models of CKD: (1) the angiotensin AT_1_ receptor antagonist losartan, and (2) the combination of the thiazide diuretic hydrochlorothiazide and the direct arteriole vasodilator hydralazine (HCTZ/HY). Both treatments have been shown to reduce BP without interacting with angiotensin II and endothelin receptors (16–20).

Here, we show for the first time that while both treatments reduced mean BP in CKD-MBD rats, both did not decrease arterial stiffness and medial vascular calcification. Therefore, our results indicate a blood pressure-independent mechanism for vascular calcification in CKD-MBD rats. Interestingly, HCTZ/HY treatment led to an unexpected aggravation of arterial stiffness and medial vascular calcification.

## Materials and methods

### Animal experiments

Male Wistar rats (Charles Rivers, Saint-Constant, Quebec, Canada), weighing about 250 g, were housed in controlled humidity and temperature conditions with a 12 h dark/light cycle and allowed free access to standard laboratory chow and tap water. All animal-related procedures were conducted per the Canadian Council on Animal Care guidelines and were approved by Université Laval's Animal Care Committee. CKD was induced by subtotal renal mass reduction, consisting of upper and lower left kidney pole resection, and right nephrectomy 1 week later (5/6 nephrectomy) performed under isoflurane anesthesia. This results in a reduction in renal function (increased serum creatinine and urea, and reduced creatinine clearance) of about 1/3 of values in normal control rats, which declined further with time as renal injury progresses, as previously reported (13–15, 17, 21). The following week, MBD was generated by a high calcium (1.2%) and phosphorus (1.2%) diet (Harlan Teklab, Madison, WI, USA), supplemented with 3 weekly subcutaneous injections of 0.5 µg/kg calcitriol (1,25-dihydroxyvitamin D3; vitamin D; Sigma-Aldrich, St. Louis, MO, USA). These conditions aim to simulate pro-calcifying conditions as seen in CKD patients and were shown to be required for the induction of medial vascular calcification and bone formation disorders in CKD rats (13–15, 21). In this study, 4 groups of animals were studied: (1) CKD rats under a standard diet that served as control without vascular calcification (*n* = 10), (2) CKD-MBD rats under calcium/phosphate-rich diet and vitamin D supplementation, which induce medial vascular calcification (*n* = 12), (3) CKD-MBD rats treated with the angiotensin II AT_1_ receptor antagonist losartan (25 mg/kg/d, in drinking water; *n* = 12), and, (4) CKD-MBD rats treated with the combination of hydrochlorothiazide and hydralazine (HCTZ/HY; 80 mg/L and 25 mg/L, respectively, in drinking water; *n* = 12). The animals were studied at week 5 due to a significant decline in the health status of CKD-MBD rats under HCTZ/HY treatment (significant weight loss and weakening appearance) reaching the ethical endpoints for euthanasia. The animals were placed in metabolic cages and 24-h urine samples were collected and stored at −20°C. Then, animals were anesthetized for the assessment of hemodynamic parameters and sacrificed by exsanguination. The thoracic aorta was harvested for histological and biochemical analyses of medial vascular calcification.

### Blood pressure and arterial stiffness assessment

Before sacrifice, animals were anesthetized with isoflurane and the right carotid artery was catheterized for the assessment of systolic BP (SBP), diastolic BP (DBP), mean BP (MBP), and pulse pressure (PP). Then, the right femoral artery was catheterized for the assessment of carotid-femoral pulse wave velocity (PWV), an index of arterial stiffness. The carotid and the femoral pulse waves were recorded and used to calculate the PWV by dividing the distance between the two catheters by the time needed for the wave to make the distance, as previously performed (13–15).

### Vascular calcification assessment

The thoracic aorta was fixed for 24 h, dehydrated, and embedded in paraffin. 5 µm thick longitudinal sections were mounted on glass slides. Tissue sections were deparaffinized and rehydrated before von Kossa staining of medial vascular calcification as previously performed (13–15, 21). Briefly, thoracic aorta sections were incubated in 5% silver nitrate for 1 h and exposed to light using a 100 W lamp, washed three times in distilled water, and placed in 5% sodium thiosulfate for 5 min. Two additional washes in distilled water were performed and samples were soaked in nuclear fast red for 2 min. Finally, thoracic aorta sections were washed three times in distilled water and dehydrated. Quantification was performed throughout all the vessel length at a magnification of 10× using an inverted microscope (Olympus, Tokyo, Japan) equipped with a CCD camera (Sony C-350) and the ImagePro-Plus analysis software (Media Cybernetics, Silver Spring, MD).

### Immunohistochemical analyses of osteocalcin and infiltration of macrophages

Thoracic aorta sections were mounted on glass slides, deparaffinized, and rehydrated. Antigen retrieval was performed by heating the tissue sections in a citrate buffer for 8 min followed by a 1 h incubation in the blocking solution containing 10% bovine serum albumin in phosphate-buffered saline. Samples were rinsed in tris-buffered saline containing 0.1% tween (TBST) and incubated overnight at 4°C with a mouse anti-rat osteocalcin to reveal vascular smooth muscle cells differentiation into osteoblast-like cells or the mouse anti-rat CD68 ED1 to assess subendothelial macrophage infiltration, as previously described (13–15, 21). Then, tissue sections were rinsed 3 times with TBST for 5 min and incubated for 15 min with a biotin-conjugated anti-mouse antibody (EMD Millipore, Billerica, MA). Horseradish peroxidase activity was detected using diaminobenzidine reagent (Thermo Scientific, Waltham, MA). Vectastain HRP ABC Reagent (Vector Laboratories, Burlingame, CA) was used for detection. Slides were counterstained with hematoxylin. Mouse isotype control antibodies were used as negative control for osteocalcin and CD68 immunohistochemical staining (Supplementary Figure S1). Quantification of specific immunostaining (brown staining color area divided by whole media area) was performed throughout the complete vessel length at 10× magnification using an inverted microscope and the ImagePro-Plus analysis software as described above.

### Sodium chloride cotransporter expression assessment

Sodium chloride cotransporter (NCC) expression was assessed in thoracic aorta sections by immunohistochemical analysis with an anti-rat NCC (SLC12A3, Abcam, Waltham, MA, USA) using the method described above. NCC expression was also determined by real-time quantitative reverse transcription-polymerase chain reaction (qRT-PCR) in total RNA extracts from a frozen thoracic aorta segment using the sense TCACCCTCCTCATCCCTTATC and the antisense GCCTTTCTCTCTTCATCCATCC probes (NCBI reference: NG_009386). Conditions for the qRT-PCR assay are described in detail elsewhere (14, 15, 21, 22). Kidney sections from normal rats were used as a positive control for NCC expression with both methods.

### Biochemical parameters assessment

Calcium, phosphate, urea, and creatinine concentrations in plasma and urine were determined using an autoanalyzer system (Ilab 1800, Lexington, MA, USA). Creatinine clearance (Ccr) was determined using the following formula: Ccr = Ucr × V/Pcr, where Ucr = urine creatinine, V = urine volume in ml/min, and Pcr = plasma creatinine.

### Data analysis

A D'Agostino–Pearson normality test was used to determine the data distribution. The results are expressed as means ± SEM if a normal distribution was found. The results are expressed as median and [25th–75th percentile] if the data did not pass the normality test. To examine the effectiveness of CKD-MBD model compared to CKD alone, we used Mann–Whitney test between these groups. To study the effectiveness of antihypertensive interventions on the CKD-MBD model we used Kruskal–Wallis followed by Dunn's tests for multiple comparisons. All statistics were done using GraphPad Prism 9 software (GraphPad Software, La Jolla, CA, USA). A *P*-value of <0.05 was considered statistically significant.

## Results

### CKD-MBD vascular calcification model

**Figure 1:**
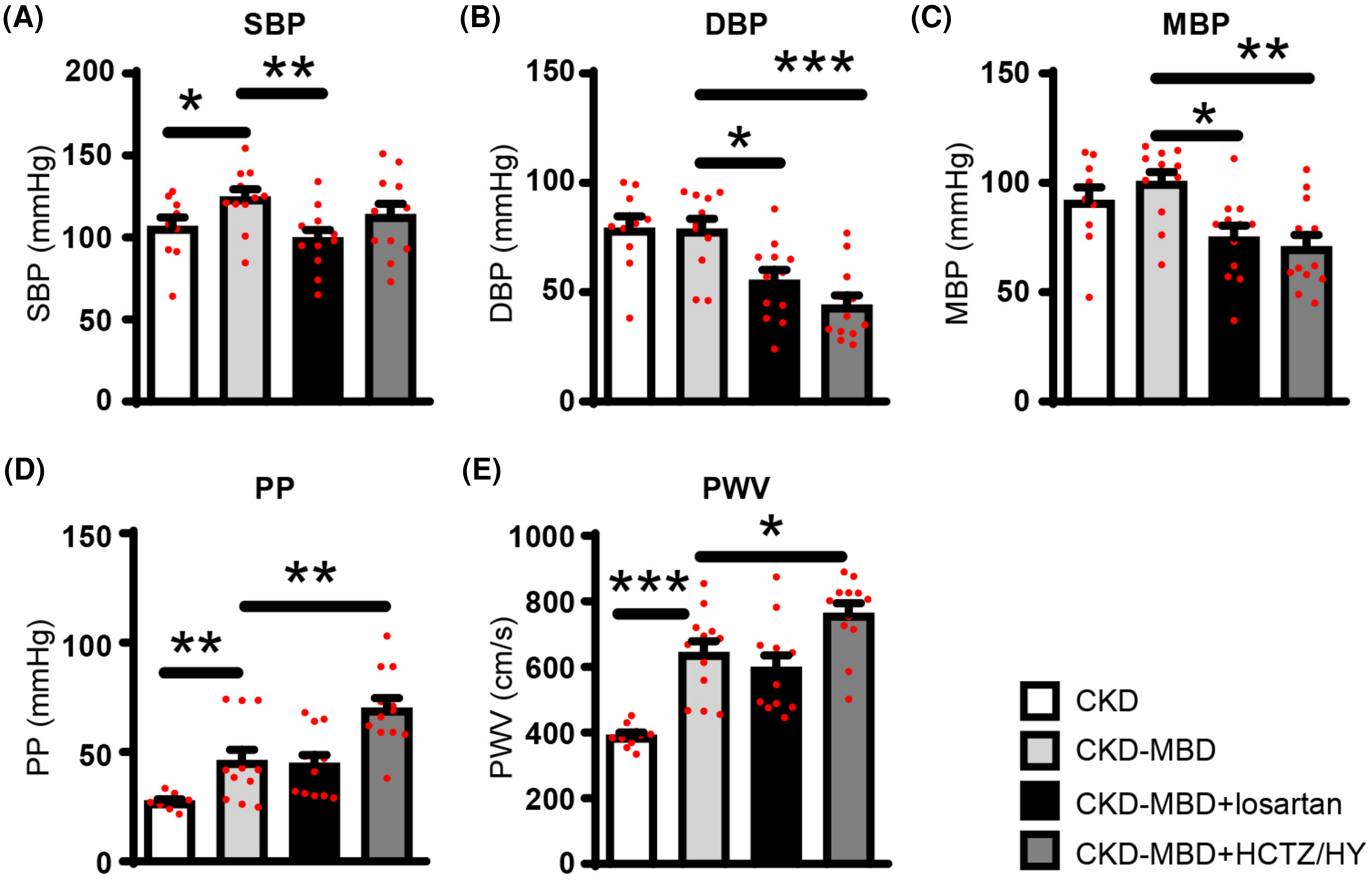
(**A**) Systolic (SBP), (**B**) diastolic (DBP), (**C**) mean blood pressure (MBP), (**D**) pulse pressures (PP), and (**E**) pulse wave velocity (PWV) assessed before animal sacrifice in control rats with chronic kidney disease (CKD; *n* = 10), in CKD rats with mineral bone disorders (CKD-MBD; *n* = 12), and CKD-MBD rats treated with losartan (*n* = 12) or the combination of hydrochlorothiazide and hydralazine (HCTZ/HY; *n* = 12). * *p* < 0.05; ** *p* < 0.01; and *** *p* < 0.001.

Table 1 shows that CKD-MBD rats had higher levels of plasma calcium and phosphate as well as increased urinary excretion of both calcium and phosphate, confirming calcium phosphate overload. As compared to the CKD rats, the CKD-MBD rats exhibited an increase in pulse pressure and carotid-femoral pulse wave velocity (Figure 1) indicating an increase in arterial stiffness. At this stage, SBP was significantly higher. On the other hand, DBP and MBP remained similar.

**Table 1:** Bodyweight, blood, and urine parameters.

Groups	CKD	CKD-MBD	CKD-MBD + losartan	CKD-MBD + HCTZ/HY
Number of rats	10	12	12	12
Bodyweight (g)	482 ± 9	449 ± 8*	445 ± 11	373 ± 11****
Plasma creatinine (µmol/L)	51.0 [46.3–57.5]	52.5 [50.3–62.8]	58.0 [50.8–65.8]	65.0 [55.3–73.8]
Plasma urea (mmol/L)	13.9 ± 0.8	12.0 ± 0.6*	13.0 ± 0.7	17.0 ± 0.7****
Plasma calcium (mmol/L)	2.58 ± 0.02	2.88 ± 0.04**	2.80 ± 0.04	2.98 ± 0.04
Plasma phosphate (mmol/L)	2.22 ± 0.07	2.59 ± 0.05**	2.74 ± 0.10	2.83 ± 0.09
Creatinine clearance (ml/min)	2.1 ± 0.1	1.8 ± 0.1	1.7 ± 0.1	1.4 ± 0.1***
Proteinuria (mg/24 h)	35 [24–55]	59 [21–109]	15 [12–20]****	25 [13–32]
Calcium excretion (mmol/24 h)	0.07 [0.03–0.09]	0.46 [0.27–0.64]**	0.31 [0.20–0.44]	0.30 [0.18–0.40]
Phosphate excretion (mmol/24 h)	0.99 ± 0.07	1.96 ± 0.12**	1.93 ± 0.06	1.52 ± 0.08****

Values are expressed as mean ± SEM or median [25th–75th percentiles]. CKD, chronic kidney disease; MBD, mineral bone disorders; HCTZ, hydrochlorothiazide; HY, hydralazine.

* *p* < 0.05.

** *p* < 0.01 vs. CKD.

*** *p* < 0.05.

**** *p *< 0.01 vs. CKD-MBD.

**Figure 2:**
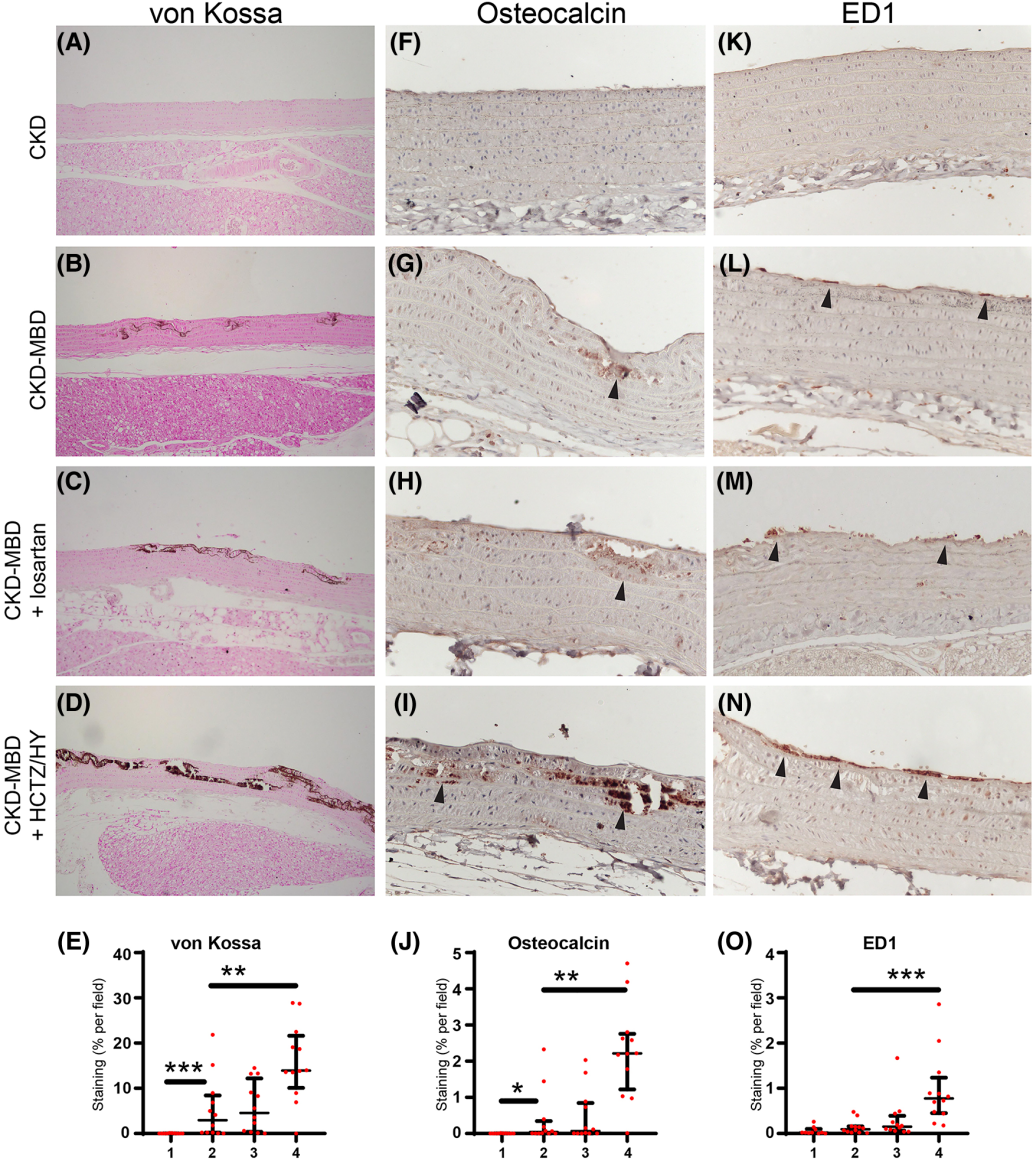
Representative photographs of medial vascular calcification assessed by von Kossa staining (brown color; **A–D**), smooth muscle cells differentiation into osteoblast-like cells by immunohistochemical staining of the bone marker osteocalcin (arrowhead; **F–I**), and inflammation by immunohistochemical staining of subendothelial macrophage infiltration using the macrophage marker CD68 antibody ED1 (arrowhead; **K–N**) in the thoracic aorta from control rats with chronic kidney disease (CKD; *n* = 10), in CKD rats with mineral bone disorders (CKD-MBD; *n* = 12), and CKD-MBD rats treated with losartan (*n* = 12) or the combination of hydrochlorothiazide and hydralazine (HCTZ/HY; *n* = 12). Quantification of von Kossa, osteocalcin, and ED1 staining are shown in the bottom line (**E,J,O**, respectively). * *p* < 0.05; ** *p* < 0.01; and *** *p* < 0.001.

Consistent with our previous studies (13–15), von Kossa staining confirms that CKD-MBD rats developed vascular calcification which was associated with significant vascular remodeling, including disruption of the elastic lamella and smooth muscle layers (Figures 2A,B,E). Thoracic aorta showed *de novo* expression of osteocalcin, a bone marker that reveals smooth muscle cells differentiation into osteoblast-like cells, in calcified areas (Figures 2F,G,J). A moderate, but not significant increase in macrophage infiltration into the subendothelial space was detected in thoracic aortas from CKD-MBD rats as compared to control CKD rats (Figures 2K,L,O).

### Impact of BP reduction by losartan on vascular stiffness and calcification

Compared to CKD-MBD rats, treatment with losartan did not have a significant effect on kidney function and mineral parameters, except for a significant reduction in proteinuria (Table 1). As expected, treatment of CKD-MBD rats with losartan significantly reduced systolic, diastolic, and mean blood pressure (Figures 1A–C). However, losartan failed to reduce pulse pressure and carotid-femoral pulse wave velocity (Figures 1D,E). Losartan treatment had no impact on the extent of vascular calcification as shown by von Kossa staining (Figures 2C,E) and *de novo* expression of osteocalcin in calcified areas was increased to a similar level as in CKD-MBD rats (Figures 2G,H,J). In keeping with the results for vascular calcification, macrophage infiltration was similar in thoracic aortas from losartan-treated CKD-MBD rats as compared to CKD-MBD rats (Figures 2L,M,O).

### Impact of BP reduction by HCTZ/HY on vascular stiffness and calcification

CKD-MBD rats treated with HCTZ/HY showed a significant decrease in body weight, associated with a decline in health status (Table 1). In addition, plasma creatinine and urea were higher in CKD-MBD rats treated with HCTZ/HY (Table 1), and the creatinine clearance was lower, indicating that HCTZ/HY treatment led to a decline in renal function. However, there were no statistically significant differences in the plasma calcium and phosphate levels along with the daily urinary excretion of calcium, while phosphate excretion was lower.

Compared to CKD-MBD rats, antihypertensive treatment with HCTZ/HY reduced DBP and MBP, but not SBP (Figures 1A–C). Unexpectedly, HCTZ/HY markedly increased pulse pressure and carotid-femoral pulse wave velocity, indicating an aggravation of arterial stiffness (Figures 1D,E). Histological analysis of the thoracic aortas revealed increased medial vascular calcification (Figure 2D). This was accompanied by a higher level of osteocalcin expression in thoracic aortas (Figure 2G,I,J) and a higher degree of macrophage infiltration (Figures 2l,N,O).

### Sodium chloride cotransporter expression

Thiazide-sensitive NCC expression has been shown in osteoblasts from human and rat bones, which was associated with cell differentiation and mineralization (23). Therefore, we used immunohistochemical and qRT-PCR analyses to determine whether NCC is expressed in calcified vessels of CKD-MBD rats and whether this expression was affected by the antihypertensive treatments. There were marked NCC staining and mRNA expression using kidney sections as positive controls. However, there was no NCC staining in the thoracic aorta nor NCC qRT-PCR amplification from any of the experimental groups.

## Discussion

In the present study, we report, for the first time, that medial vascular calcification in CKD rats with MBD is related to blood pressure-independent mechanisms. Hence, the two antihypertensive treatments used in CKD-MBD rats, including the angiotensin AT_1_ receptor antagonist losartan or the combination of hydrochlorothiazide and hydralazine failed to reduce vascular stiffness and medial vascular calcification. Although vascular calcification in CKD-MBD rats was unaffected by losartan, treatment with HCTZ/HY, unexpectedly, aggravated arterial stiffness and medial vascular calcification that were associated with a decline in renal function and health status of this group of rats.

A key finding of this study is that the treatment with losartan did not modify vascular stiffness and medial vascular calcification in CKD-MBD rats, despite a significant reduction in systolic, diastolic, and mean blood pressure. This indicates that the development of medial vascular calcification in these experimental conditions is likely related to blood pressure-independent mechanisms. In addition, losartan failed to reduce vascular smooth muscle cell differentiation into osteoblast-like cells and macrophage infiltration-related inflammation. This suggests that angiotensin II does not play a dominant role in this model of medial vascular calcification. Although angiotensin II was shown to be involved in intimal vascular calcification related to atherosclerosis, the involvement of angiotensin II in medial calcification remains controversial (6, 24). For instance, renin-angiotensin system blockade did not suppress medial vascular calcification in CKD rats on a high-phosphate diet for 4 months (25). In a non-CKD model of elastocalcinosis induced by warfarin and vitamin K_1_ treatment in rats, long-term treatment with the AT_1_ receptor antagonist irbesartan was effective in preventing vascular calcification, but was of limited effectiveness in preventing the progression of vascular calcification despite a reduction in vascular remodeling (26). In contrast, endothelin ET_A_ receptor blockade caused a significant reduction of both vascular remodeling and calcification in the latter rat model. This observation is in keeping with our previous findings showing that ET_A_ receptor blockade in CKD-MBD rats also reduced systolic blood pressure together with arterial stiffness and medial vascular calcification (15). Based on the results of the present study, the protective effects of ET_A_ receptor blockade are likely independent of the blood pressure-lowering effects. Indeed, this is supported by the beneficial effects of ET_A_ receptor blockade on mechanisms of vascular calcification such as vascular remodeling, smooth muscle cell differentiation into osteoblast-like cells, and inflammation in CKD-MBD rats (15).

Unexpectedly, treatment of CKD-MBD rats with HTCZ/HY markedly increased arterial stiffness and medial vascular calcification despite a similar reduction in mean blood pressure as compared to losartan treatment. The detrimental effect of HTCZ/HY on medial vascular calcification in CKD-MBD rats was associated with higher levels of osteocalcin expression and ED1 immunostaining, indicating increased vascular smooth muscle cell differentiation into osteoblast-like cells and subendothelial macrophage infiltration-related inflammation, respectively. Although there is no evidence suggesting the involvement of HY in the latter pathological effects, several studies support a possible role for thiazide diuretics, such as HCTZ, in vascular calcification. While the thiazide-sensitive NCC is not normally expressed in vascular smooth muscle cells (27), NCC expression was shown in osteoblasts from human and rat bones (23). For instance, NCC inactivation by thiazide diuretics induces osteoblast differentiation marker expression including runt-related transcription factor 2 (Runx2) and osteopontin, and mineralization that is associated with increased bone density (23). Similarly, NCC gene inactivation led to osteoblast differentiation and calcium deposition in mice (28). Therefore, we investigated the possibility that the thiazide diuretic HCTZ may aggravate vascular calcification in CKD-MBD rats through NCC activity in differentiated vascular smooth muscle cells. In the present study, we did not find NCC expression in thoracic aortas in all experimental groups, suggesting an indirect effect of HCTZ on vascular calcification. One possibility is that the adverse effect of HCTZ may be related to the worsening renal function associated with greater phosphate retention, a major determinant of vascular calcification (29). This renal pathological phenomenon may also be related, at least in part, to HCTZ-induced apoptosis of distal tubule cells, as well as peritubular and vascular inflammation that could aggravate renal injury (30–32). On the other hand, HY treatment neither prevented nor aggravated renal injury in CKD rats despite a significant reduction in blood pressure, suggesting a minor contribution of HY (33). The loss of renal function together with increased arterial stiffness and medial vascular calcification induced by HCTZ/HY treatment are major morbidity factors that certainly contributed to the significant decline in the health status of CKD-MBD rats. Another possibility is that HCTZ, through activation of renin-angiotensin system, could lead to a rise in plasma aldosterone (34), which was reported to play a major role in vascular calcification (35). Indeed, aldosterone increased the expression of type III sodium-dependent phosphate transporter Pit1 thereby increasing intracellular phosphate levels that promoted vascular osteoinduction and calcification (36, 37). While the renin-angiotensin system is a key regulator of aldosterone secretion from the adrenal glands, angiotensin II AT1 receptor blockade with losartan did not show a beneficial effect on medial vascular calcification in CKD-MBD rats, suggesting that its involvement is limited. Alternative mechanisms of aldosterone synthesis might be activated in pro-calcifying conditions such as in CKD-MBD rats. For instance, vascular smooth muscle cells can produce aldosterone through the upregulation of the aldosterone synthase CYP11B2 expression in high phosphate conditions thereby inducing osteogenic transformation (38). Moreover, upregulation of aldosterone synthase CYP11B2 expression in adrenal glands can be induced by klotho deficiency, which also occurs in CKD and has been associated with vascular calcification (39, 40). Given that the thiazide diuretic HCTZ is largely used for the management of blood pressure in subjects with cardiovascular disease including CKD patients, our findings may have significant clinical implications. However, our study cannot clearly distinguish if the cause is HCTZ, hydralazine, or the combination of both compounds for the aggravation of medial vascular calcification and will require further investigation.

In conclusion, in the rat remnant kidney model of CKD with MBD, antihypertensive treatment with the angiotensin AT_1_ receptor antagonist losartan had no significant impact on arterial stiffness and medial vascular calcification. Unexpectedly, HCTZ/HY treatment aggravated arterial stiffness and vascular calcification despite a reduction in mean blood pressure, suggesting blood pressure-independent mechanisms of vascular calcification.

## Data Availability

The raw data supporting the conclusions of this article will be made available by the authors, without undue reservation.
