# Supplementary figures and images for: Antihypertensive treatment with hydrochlorothiazide-hydralazine combination aggravates medial vascular calcification in CKD rats with mineral bone disorder
Source: Front Cardiovasc Med. 2023 Sep 29;10:1241943. doi: 10.3389/fcvm.2023.1241943 (PMC10570511; doi:10.3389/fcvm.2023.1241943)

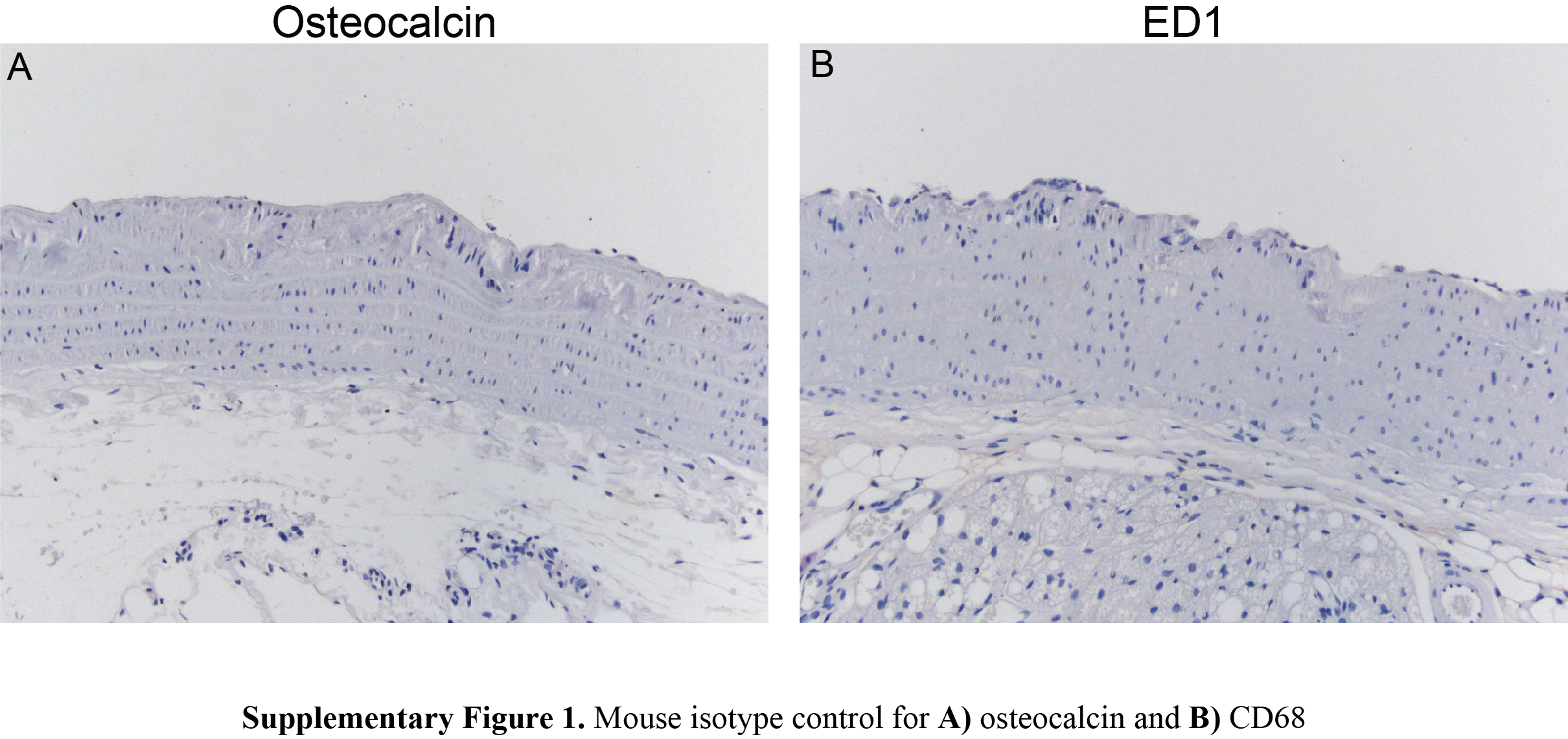

Supplement: Supplementary file 1 [file Image1.jpeg]
